# Water Extract of* Acori Graminei* Rhizoma Attenuates Features of Rheumatoid Arthritis in DBA/1 Mice

**DOI:** 10.1155/2019/3637453

**Published:** 2019-01-01

**Authors:** Jong-Hyun Nho, A-Hyeon Kim, Ho-Kyung Jung, Mu-Jin Lee, Ji-Hun Jang, Beo-Dul Yang, Hyun-Ju Lee, Ki-Ho Lee, Kyeong-Wan Woo, Hyun-Woo Cho

**Affiliations:** National Development Institute of Korean Medicine, Jangheung-gun 59338, Republic of Korea

## Abstract

The dry rhizome of* Acorus gramineus *Solander, known as* Acori Graminei* Rhizoma, is used to treat dementia, stroke, eczema, and indigestion in traditional Chinese medicine, traditional Korean medicine, and traditional Japanese Kampo medicine. Previous studies have reported that* Acori Graminei* Rhizoma extract ameliorated cognitive impairment in A*β*1-42 injected mice. However, the effect of* Acori Graminei* Rhizoma on type II collagen induced arthritis (CIA) has not been elucidated. Thus, we evaluated the water extract of* Acori Graminei* Rhizoma (WAG) in CIA mice models. Male DBA/1 mice were separated into five groups (NOR; n=10, CON; n=10, CIA + methotrexate (MTX); n=10, CIA + 100 mg/kg WAG; n=10, CIA + 500 mg/kg WAG; n=10). CIA was induced by injecting the mice with bovine type II collagen, after which the mice were treated with WAG and/or MTX. Hematological parameters and liver and kidney serum toxicity markers were analyzed. Further, serum levels of interleukin (IL)-6, TNF-*α*, and type II collagen IgG were analyzed via enzyme-linked immunosorbent assay (ELISA). Treatment with 500 mg/kg WAG decreased serum levels of IL-6, TNF-*α*, and collagen IgG in a CIA model. Moreover, WAG treatment decreased CIA-induced swelling of mouse hind legs, infiltration of inflammatory cells into the synovial membrane, and blood neutrophil levels. WAG administration did not influence hematological parameters or kidneys and liver toxicity markers. WAG may be used to treat arthritis by reducing the inflammation indicators. However, further experiments are required to determine how WAG affects inflammation mechanisms* in vitro* and* in vivo*.

## 1. Introduction

*Acori Graminei* Rhizoma, the rhizome of* Acorus gramineus *Solander, is commonly found in South Korea, China, and Japan.* Acori Graminei* Rhizoma has been shown to inhibit microglial neuroinflammation and neurotoxicity in an MPTP (1-methyl-4-phenyl-1,2,3,6,-tetrahydropyridine)-induced mouse model of Parkinson's disease and to diminish contractile dysfunction in an ischemic and reperfused rat heart [1, 2]. Moreover, treatment with essential oil derived from* Acori Graminei* Rhizoma improved learning and memory in aged rats and mice [3]. In traditional Chinese and Korean medicine,* Acori Graminei* Rhizoma is used as a therapeutic agent for dementia, stroke, eczema, and indigestion [4]. Previous studies of* Acori Graminei* rhizome reported some phenolic compound including *α*-asarone and *β*-asarone, which were closely associated with anti-inflammatory effect [5–7]. According to report, the essential oils of the rhizomes of* Acori Graminei* contained various chemical compounds including *β*-asarone (43%) as the major compound, cis-asarone, acortatarins A, calamenone, chrysophanol, and emodin [8]. Emodin has antirheumatic effect on CIA models [9], and chrysophanol has anti-inflammatory activity through the suppression of NF-kappaB/caspase-1 activation [10]. *β*-asarone attenuated TNF-alpha in lipopolysaccharide-stimulated cells [11]. *α*-asarone decreased TNF-alpha decreasing NF-kappaB activation in pilocarpine-induced status epilepticus [12]. TNF-*α* is a proinflammatory cytokine which mediated rheumatoid arthritis (RA) pathogenesis (Vasanthi et al., 2007). Effect of many compounds including *α*-asarone, *β*-asarone, cis-asarone, and acortatarins A on CIA models has not been studied. Despite this, the effect of water extract from* Acori Graminei* Rhizoma on type II collagen induced arthritis (CIA) has not been elucidated. For these reasons, we investigated the effect of* Acori Graminei* Rhizoma water extract (WAG) on CIA in an animal model.

RA is an inflammatory and autoimmune disease that primarily affects the synovial membrane and the joint in the hind legs. It is characterized by the presence of rheumatoid factors (RF), such as autoantibodies to serum IgG (Immunoglobulin G). Over time, RA can lead to joint destruction and irreversible handicap [13]. Known genetic risk factors for RA include anticitrullinated protein antibodies (ACPAs), specific class II human leukocyte antigen (HLA as a major histocompatibility complex), Dickkopf-related protein (DKK1), and matrix metallopeptidase 9 (MMP-9), whereas known nongenetic risk factors include smoking, microbiota, and female sex [14–18]. While the pathophysiological mechanisms of RA remain unclear, it is known that various inflammatory factors such as B- and T-lymphocytes, fibroblast-like synoviocytes, and chondrocytes are involved in RA pathogenesis [19, 20]. The CIA model is an animal model that has commonly been used to study RA. CIA treatment in animals causes the development of RA-like characteristics including swelling and erythema of the hind legs, an inflammatory response in the synovial membrane and joint, and elevated type II collagen IgG in the serum [21].

In recent years, the World Health Organization has reported that the use of complementary and alternative medicines to prevent disease has been increasing worldwide [22]. In traditional Korean medicine, various medicinal plants are used to treat disease. However, an overdose of extracts from medicinal plants can induce cytotoxicity [23]. For example, one study showed that ginseng (Pana spp.) may have toxic effects such as hypertension [24]. Moreover, overdoses of various medicinal plant extracts have induced hepatotoxicity and renal failure [25, 26]. Thus, we examined changes in hematological indicators and toxicity markers for the liver and kidney.

## 2. Method and Materials

### 2.1. Samples and Extraction

*Acori Graminei* Rhizoma was purchased in the Seoul traditional market kyungdong sijang (Seoul, Korea). It was taxonomically identified by Jong Gil Jeong (Plant taxologist, Dongshin University, Naju, Korea). The identified positive sample of* Acori Graminei* Rhizoma (TKMII-3) was stored at the National Development Institute of Korean Medicine (NIKOM, Jangheung, Korea). In order to prepare the extract,* Acori Graminei *Rhizoma was washed with sterilized water and dried at 50°C for 1 week using the hot air drying oven (SAM-DR1700, Samgongsa, Gyeonggi, Korea). In order to prepare the extract,* Acori Graminei *Rhizoma was washed with sterilized water and dried at 50°C for 1 week using the hot air drying oven (SAM-DR1700, Samgongsa, Gyeonggi, Korea). The resultant dried A*cori Graminei *Rhizoma (2450 g) was pulverized using an electronic mixing machine and extracted with water (30 L, 3 times) under reflux conditions for 3 hr. The extract was then filtered through 15 cm fluted qualitative filter paper circles (Thermo, Waltham, MA, USA) and lyophilized using a freeze dryer (LYOPH-PRIDE 20R, IlShinBioBase, Dongducheon, Korea) and obtained WAG powder (593 g, yield: 24%). The lyophilized powder was dissolved in phosphate buffered saline (PBS, pH 7.4) prior to use. The lyophilized powder dissolved with 0.5% carboxymethyl cellulose (CMC, Sigma Aldrich, USA), used to experiment.

### 2.2. LC-IT-TOF-MS Conditions of Acori Graminei Rhizoma Extract

WAG was analyzed by ESI-mass spectra which were obtained on a LC-IT-TOF mass spectrometer (Shimadzu, Japan). Solvents for samples analysis were HPLC grade, purchased from JT Baker (PA, USA). The Shimadzu UFLC (Kyoto, Japan) system used for analysis consisted of two LC-30AD XR pumps, a CTO-20A column oven, a SPD-20A detector, a SIL-20A XR automatic injector, and a DGU-20A3 degasser. The MS scan was measured at* m/z* 100-1000. Data processing was done by UFLC and MS, and LCMS solution software (version 3, Shimadzu, Japan) was used. For component analysis of WAG using LC/MS, the analysis conditions are set as shown in Table 1.

### 2.3. Preparation of Standard and Sample Solution

*β*-Asarone (d=1.073 g/mL, purity 70%) purchased from Sigma Aldrich (St. Louis, MO, USA) was prepared by dissolving the reference standard in methanol to final concentration of 751.1 *μ*g/mL and diluted to obtain concentrations at 300.44, 120.176, 48.0704, 24.0352, and 12.0176 *μ*g/mL. The powder of WAG (150 mg) was extracted with 45 mL of 0%, 30%, 50%, 70%, and 100% methanol, respectively, sonicated for 60 min at room temperature. Each extract was filtered in a volumetric flask; extract solution was adjusted to a final volume of 50 mL. The extraction efficiency of each solvent was analyzed three times. The content analysis according to extraction methods (sonication and reflux) was weighing in the same way and then extracted and used as the solvent with the best extraction efficiency. The extracts were filtered through 0.2 *μ*m syringe filter and used [27–29].

### 2.4. Quantitative Analysis

The content of *β*-asarone in extract was calculated using its calibration curve. The calibration curve was prepared using the peak area according to the concentration using the standard solution prepared. The quantitative analysis was performed on a LC-20AD HPLC system (Shimadzu, Japan), equipped with two LC-20AD (binary) pumps, a SPD-M20A detector (diode array detector), and a SIL-20A automatic injector. A SunFire C18 column (5 *μ*m, 4.6 × 250 mm, Waters) was used for the analysis. The mobile phase was composed of 0.1% formic acid in water (A) and 0.1% formic acid in acetonitrile (B) at 35°C. The gradient elution was 40% B to 60% B for 0-25 min, constant at 60% B for 5 min, 60% B to 40% B for 8 min. The analysis was performed at a flow rate 1.0 mL/min with the wavelength set at 254 nm [30, 31].

### 2.5. Animals and Treatment

Male DBA/1 mice (6 weeks old), purchased from Orient Bio (Jeongeup, Republic of Korea), were separated into five groups (NOR; n=10, control (CON); n=10, CIA + methotrexate (MTX); n=10, CIA + 100 mg/kg WAG; n=10, CIA + 500 mg/kg WAG; n=10). Bovine type II collagen (Immunization grade, Chondrex, Redmond, WA, USA) was dissolved in complete Freund's adjuvant (CFA, Sigma Aldrich, St. Louis, MO, USA) or in incomplete Freund's adjuvant (IFA, Sigma Aldrich, St. Louis, MO, USA). First, mice were injected with bovine type II collagen dissolved in CFA (1:1 ratio) into the base of the tail (100 *μ*L). One week later, 100 *μ*L bovine type II collagen was dissolved in IFA (1:1 ratio) and injected into the base of the tail [32]. After 1 week, the CIA + 100 mg/kg WAG group was treated with 100 mg/kg WAG (p.o., once a day, 3 weeks). The CIA + 500 mg/kg WAG group was treated with 500 mg/kg WAG (p.o., once a day, 3 weeks). The CIA + MTX group was treated with methotrexate (0.2 mg/kg, p.o., once a day, 3 weeks).

### 2.6. Clinical Assessment of Arthritis

Arthritis scoring was performed following the procedure explained by Endale et al. Clinical assessment was as follows: 0 = symptomless, 1 = erythema, 2 = erythema and mild swelling, 3 = erythema from the tarsals to the ankle and mild swelling, 4 = erythema from the metatarsal joints to the ankle and moderate swelling, and 5 = erythema from the foot to the ankle and severe swelling [33]. Scoring was performed by three independent investigators and was performed weekly, beginning from 21 days after the second inoculation.

### 2.7. Enzyme-Linked Immunosorbent Assay (ELISA)

ELISA was conducted for measurement of IL-6 and TNF-alpha in serum using Mouse TNF-*α* DuoSet enzyme-linked immunosorbent assay (ELISA; DY410-05, R&D systems, MN, USA) and Mouse IL-6 DuoSet ELISA (DY406-05, R&D systems, MN, USA), according to the manufacturer's instructions. Briefly, blood was collected in BD Vacutainer™ SST tubes (Thermo, MA, USA) and incubated at room temperature for 20 minutes. The tubes were then centrifuged for 10 minutes at 4,000 rpm at 4°C, and the serum was extracted to evaluate TNF-*α* and IL-6.

### 2.8. Measurement of Type II Collagen IgG

Serum measurements of type II collagen IgG were performed using a Mouse anti-mouse type II collagen IgG antibody assay (2036, Chondrex, WA, USA), according to the manufacturer's instructions. Briefly, whole blood was collected in BD Vacutainer™ SST tubes and incubated at room temperature for 10 minutes. The tubes were then centrifuged for 10 minutes at 4,000 rpm at 4°C, and the serum was extracted to determine type II collagen IgG levels.

### 2.9. Hematoxylin & Eosin (H&E) Staining

Hind limbs were harvested from the mice, fixed in 10% neutralized buffered formalin (NBF, Sigma Aldrich, St. Louis, MO, USA) for 24 hours and paraffinized with paraplast (39603002, LEICA biosystems, Wetzlar, Germany), diluted ethanol, and xylene (Sigma Aldrich, 534056, St. Louis, MO, USA). Paraffinized hind limb samples were cut into 5 *μ*m slices using a microtome (LEICA biosystems, Wetzlar, Germany), deparaffinized with xylene, and hydrated with diluted ethanol and water. Deparaffinized sections were subjected to H&E staining (Sigma Aldrich).

### 2.10. Blood Chemistry Analysis

Blood chemistry was analyzed using a FUJI DRI-CHEM 4000i analyzer (Fujifilm, Tokyo, Japan), according to the manufacturer's instructions. Briefly, whole blood was collected in BD Vacutainer™ SST tubes and incubated at room temperature for 10 minutes. The samples were then centrifuged for 10 minutes at 4,000 rpm at 4°C, and the serum was removed for blood chemistry analysis (blood urine nitrogen, BUN; creatinine, Cre; aspartate serum transferase, AST; alanine amino transferase, ALT).

### 2.11. Hematological Analysis

The analysis of whole blood cells was conducted using an IDEXX ProCyte DX hematology analyzer (IDEXX, Westbrook, ME, USA), according to the manufacturer's instructions. Briefly, whole blood harvested from mice was collected in BD Vactutainer™ glass blood collection tubes with K_3_ EDTA (Thermo, MA, USA) and then used for analysis. Imaging resulted in the coloring of cells as follows: blue for lymphocytes, pink for neutrophils, and red for monocytes. Hematological parameters [red blood cells (RBC), hemoglobin (HGB), hematocrit (Hct), mean corpuscular volume (MCV), mean corpuscular hemoglobin (MCH), and mean corpuscular hemoglobin concentration (MCHC)] were presented in a graph.

### 2.12. Statistical Analysis

Results were expressed as mean ± SEM. Between groups comparisons were conducted using one-way ANOVA by SPSS (SPSS Inc., IL, USA), followed by Tukey post hoc test. A value of* p* < 0.05 was considered significant. We compared groups on rheumatoid arthritis score using one-way ANOVA with Tukey-Kramer multiple comparison test. P value was less than 0.05.

## 3. Results

### 3.1. LC-IT-TOF-MS Conditions of Acori Graminei Rhizoma Extract

**Figure 1:**
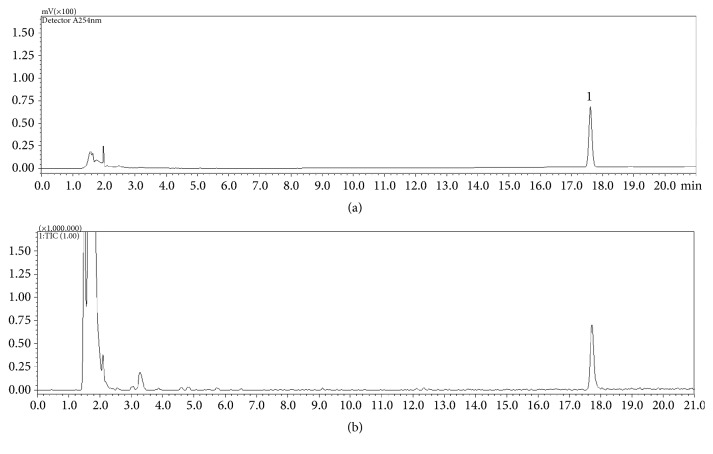
LC-IT-TOF MS chromatograms of* Acori Graminei* Rhizoma. LC chromatogram (a) and total ion chromatogram in positive ion mode (b). *β*-asarone (1).

As shown in Figure 1, the IT-TOF-MS analysis of WAG showed one peak at 17.63 min and the molecular ion peak of m/z 209.12 [M+H]^+^ was confirmed in positive mode. Based on the literature, it was confirmed that the retention time and the molecular weight of the *β*-asarone standard obtained from sigma were in agreement with each other [28, 34].

### 3.2. Quantitative Analysis

**Figure 2:**
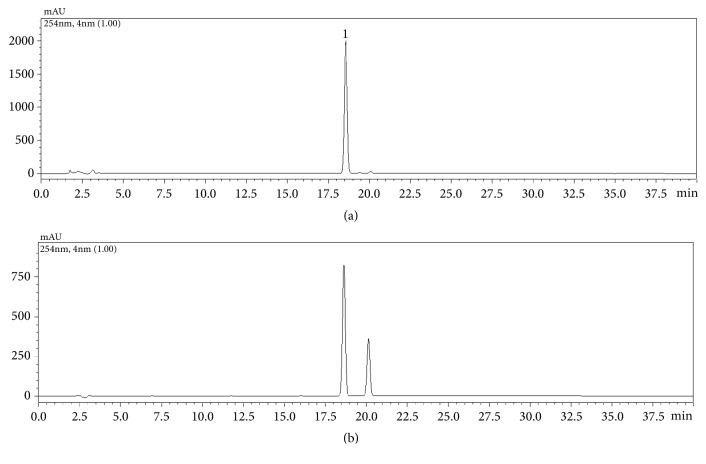
HPLC chromatogram of WAG (a) and standard solution (b). *β*-asarone (1).

The comparison chromatogram of WAG and standard was confirmed in Figure 2. The calibration curve was prepared for *β*-asarone, which is a major compound of WAG. The calibration curve correlation coefficient (r^2^) in the range of 12.0176-300.44 *μ*g/mL was 0.999, indicating a good linearity (Table 2). Based on the comparison of the content by extraction solvent of 0%, 30%, 50%, 70%, and 100% methanol, the highest extraction efficiency of 30% methanol was found (Table 3). Therefore, 30% methanol was used for the overall content analysis experiment. Comparing the result of the content analysis for the two extraction methods (sonication extraction and reflux extraction) resulted in a higher content of compound when sonication extraction was used. For optimization of sample preparation conditions, sample was extracted for 60, 120, and 180 min using 30% methanol and the sonication extraction. As a result of comparing the content of compound according to the extraction time, when the extraction time was 60 min, content of compound was lower by a small difference than in other extract, but relative standard deviation was the best. In conclusion in this experiment, *β*-asarone which is a major compound of WAG showed excellent efficiency when sonication extracted with 30% methanol for 60 min, and the content of WAG was 15.32±0.26 mg/g.

### 3.3. WAG Administration Diminished CIA-Induced Swelling and Erythema of the Hind Limb

To examine the effect of WAG administration in a CIA model, changes to the swelling and erythema of the hind limb were examined. In addition, experimental schedule indicated diagram (Figure 4(a)). Treatment with 500 mg/kg WAG significantly reduced the amount of swelling and erythema relative to the CON group (CON, 8 + 0.5; CON + MTX, 4.5 + 0.7; CIA + 100 mg/kg WAG, 7.0 + 1.0; CON + 500 mg/kg WAG, 5.5 + 0.5), although it was not more efficient than 0.2 mg/kg MTX treatment (Figures 4(b) and 4(c)). Nonetheless, 500 mg/kg WAG statistically reduced erythema and swelling of hind limb compared with CON groups.

### 3.4. Effect of WAG Administration on Neutrophilia and the Infiltration of Inflammatory Cell into the Synovial Membrane

To further elucidate the effect of WAG in a CIA animal model, we examined its effect on neutrophil levels via histological staining and serum levels. As shown in Figures 2(a) and 2(b), neutrophil serum levels increased in CIA groups. However, these levels decreased with treatment of 0.2 mg/kg MTX, 100 mg/kg WAG, or 500 mg/kg WAG (NOR, 15.50 + 0.72%; CON, 56.00 + 1.34%; CON + MTX, 19.80 + 2.10; CON + 100 mg/kg WAG, 27.03 + 1.00; CON + 500 mg/kg WAG, 23.50 + 0.72). Moreover, the infiltration of inflammatory cells into the synovial membrane (black arrowhead) decreased after WAG administration to the synovial membrane and knee joints (Figure 4(c)). These data suggest that treatment with WAG ameliorated the infiltration of inflammatory cells and increased serum neutrophil levels in a CIA animal model.

### 3.5. WAG Treatment Reduced Serum Levels of IL-6, TNF-*α*, and Type II Collagen IgG

**Figure 3:**
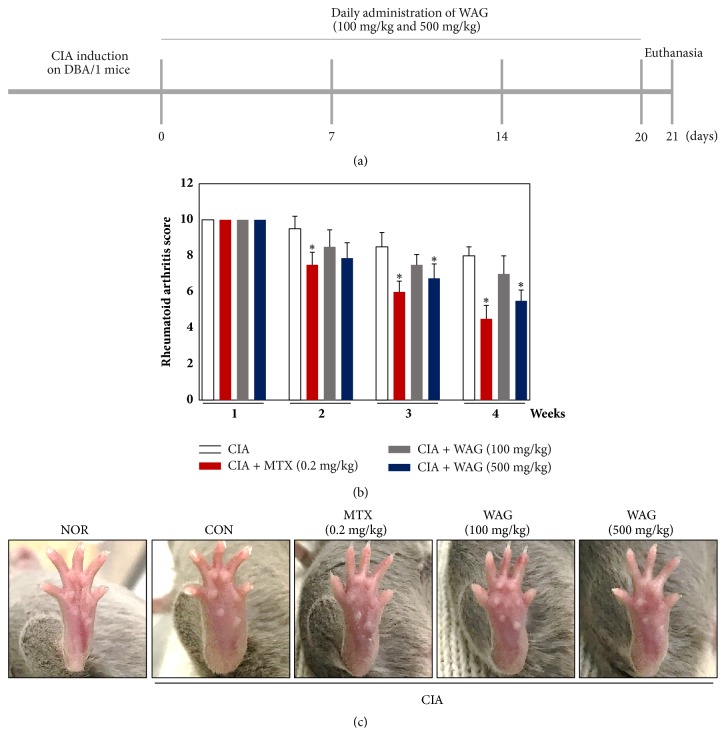
WAG administration diminished CIA-induced swelling of hind legs. (a-c) Mice were separated into five groups (NOR; n=10, Control (CIA); n=10, 0.2 mg/kg MTX; n=10, 100 mg/kg WAG; n=10, 500 mg.kg WAG; n=10). After CIA induction, mice were administered with WAG (100 or 500 mg/kg). Morphological analysis was carried out on hind legs. Rheumatoid arthritis score was assessed weekly beginning from 21 days of immunization. They were examined for four times per week. Clinical assessment is as follows: 0 = symptomless, 2 = erythema, 4 = mild swelling and erythema, 6 = mild swelling, erythema from the tarsals to the ankle, 8 = moderate swelling, erythema from the metatarsal joints to the ankle, and 10 = severe swelling and erythema from the foot to the ankle. Means with difference letters are significantly different at ^*∗*^*p* < 0.05* vs*. CIA group by one-way ANOVA with Tukey-Kramer multiple comparison test, CIA, collagen induced arthritis; MTX, methotrexate; WAG, water extracts of* Acori Graminei* Rhizoma.

**Figure 4:**
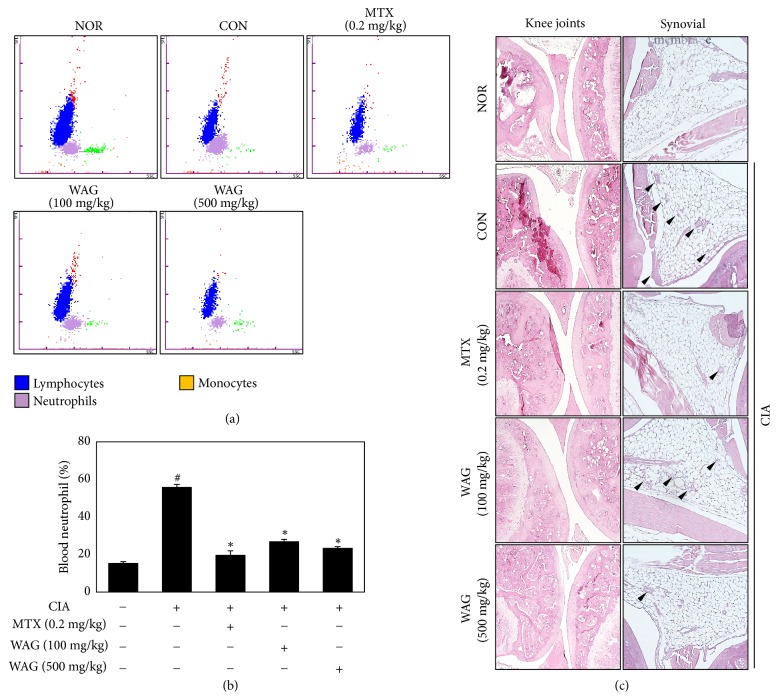
Effect of WAG administration on the level of blood neutrophil and infiltration of inflammatory cell in synovial membrane. Mice were separated into five groups (NOR; n=10, Control (CIA); n=10, 0.2 mg/kg MTX; n=10, 100 mg/kg WAG; n=10, 500 mg.kg WAG; n=10). (a-b) Blood neutrophils were analyzed by IDEXX ProCyte. Data represent the mean ± SEM of three independent experiments. (c) Representative images were stained by hematoxylin and eosin (H&E) staining; infiltration of inflammatory cell was indicated with black arrowhead. Means with difference letters are significantly different at ^#^*p* < 0.05* vs*. normal group; ^*∗*^*p* < 0.05* vs*. CIA group by one-way ANOVA with Tukey post hoc test. CIA, collagen induced arthritis; MTX, methotrexate; WAG, water extracts of* Acori Graminei* Rhizoma.

**Figure 5:**
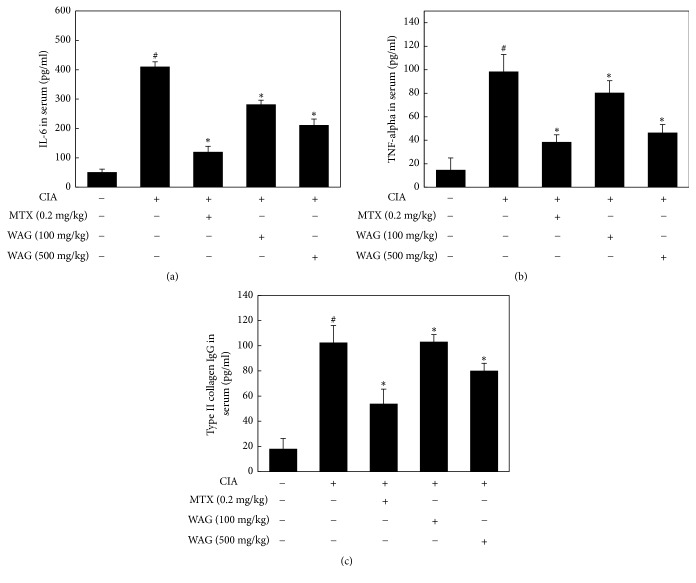
Effect of WAG on CIA-induced induction of inflammatory cytokines and type II collagen IgG in serum. Mice were separated into five groups (NOR; n=10, Control (CIA); n=10, 0.2 mg/kg MTX; n=10, 100 mg/kg WAG; n=10, 500 mg.kg WAG; n=10). (a-c) IL-6, TNF-alpha, and type II collagen IgG were analyzed by ELISA assay. Means with difference letters are significantly different at ^#^*p* < 0.05* vs*. normal group; ^*∗*^*p* < 0.05* vs*. CIA group by one-way ANOVA with Tukey post hoc test. CIA, collagen induced arthritis; MTX, methotrexate; WAG, water extracts of* Acori Graminei* Rhizoma.

To examine the antirheumatic effect of WAG treatment* in vivo*, WAG was orally administered to CIA-induced mice and then serum levels of IL-6, TNF-*α*, and type II collagen IgG were evaluated. The administration of 500 mg/kg WAG ameliorated the elevated IL-6 levels caused by CIA induction (Figure 4(a)), although this effect was not stronger than that of MTX (NOR, 51.31±10.12 pg/mg; CON, 410.51±16.54 pg/mg; CON + MTX, 120.51±18.51 pg/mg; CON + 100 mg/kg WAG, 282.21±13.84 pg/mg; CON + 500 mg/kg WAG, 211.54±20.41 pg/mg). As shown in Figure 3(b), increased TNF-*α* serum levels decreased after treatment with 500 mg/kg WAG (NOR, 16.75±13.46 pg/mg; CON, 101.76±21.81 pg/mg; CON + MTX, 39.66±4.44 pg/mg; CON + 100 mg/kg WAG, 77.73±6.79 pg/mg; CON + 500 mg/kg WAG, 48.75±4.27 pg/mg). Moreover, WAG treatment significantly reduced type II collagen IgG serum levels relative to those in CON groups (NOR, 18.14±8.15 pg/mg; CON, 102.51±13.57 pg/mg; CON + MTX, 53.92±11.61 pg/mg; CON + 100 mg/kg WAG, 103.14±5.76 pg/mg; CON + 500 mg/kg WAG, 80.14±5.88 pg/mg). However, WAG administration only weakly affected type II collagen IgG levels previously increased by CIA administration (Figure 5(c)). The results indicated that 500 mg/kg SCW administration was not more effective than 0.2 mg/kg MTX administration, even while it did affect the anti-inflammatory response in a CIA animal model.

### 3.6. WAG Administration Did Not Influence Liver and Kidney Toxicity Markers and Hematological Parameters

**Figure 6:**
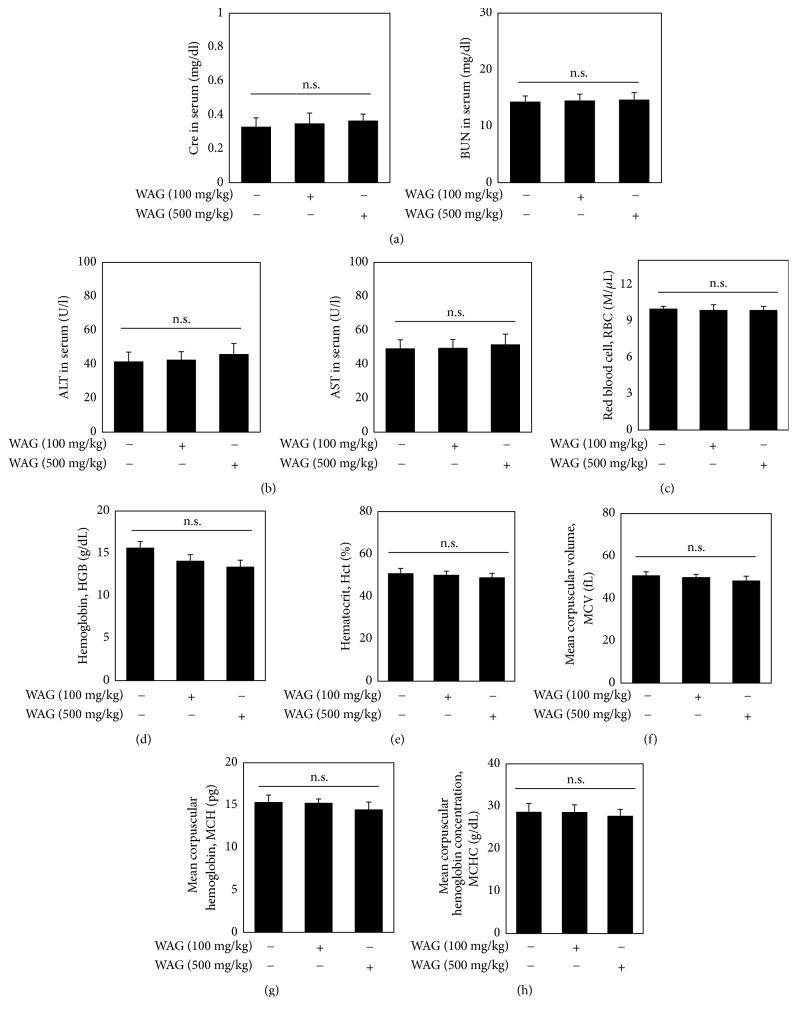
Toxicity and hematological evaluation of WAG administration on toxicity markers of kidneys, liver, and blood. (a-b) Mice were separated into three groups (NOR; n=10, 100 mg/kg WAG; n=10, 500 mg.kg WAG; n=10). Mice were administered with WAG (100 or 500 mg/kg). BUN, Cre, AST, and ALT were analyzed by FUGI DRI-CHEM 4000i. (c-h) Hematological parameters including RBC (red blood cell), HGB (hemoglobin), Hct (hematocrit), MCV (mean corpuscular volume), MCH (mean corpuscular hemoglobin), and MCHC (mean corpuscular hemoglobin concentration) were analyzed by IDEXX ProCyte. Data represent the mean ± SEM of three independent experiments. Means with difference letters are significantly different at ^#^*p* < 0.05* vs*. normal group; ^*∗*^*p* < 0.05* vs*. CIA group by one-way ANOVA with Tukey post hoc test. CIA, collagen induced arthritis; MTX, methotrexate; WAG, water extracts of* Acori Graminei* Rhizoma.

BUN and Cre are commonly used as indicators of kidney function, while ALT and AST are used as indicators of liver function [35]. In this study, we used these indicators to investigate the effect of WAG administration on the liver and kidney (Figures 6(a) and 6(b)). The results showed that WAG administration did not affect Cre serum levels (NOR, 0.33±0.05 mg/dL; 100 mg/kg WAG, 0.35±0.06 mg/dL; 500 mg/kg WAG, 0.36±0.03 mg/dL). Moreover, it was an influence on BUN in serum (NOR, 14.33±1.00 mg/dL; 100 mg/kg WAG, 14.53±1.10 mg/dL; 500 mg/kg WAG, 14.66±1.25 mg/dL). Next, we confirmed that ALT serum levels were not changed by WAG administration (NOR, 41.66±5.50 U/L; 100 mg/kg WAG, 42.35±4.72 U/L; 500 mg/kg WAG, 46.32±6.24 U/L). Similarly, as shown in Figure 4(b), serum AST levels were not influenced by WAG administration (NOR, 49.33±5.13 U/L; 100 mg/kg WAG, 49.66±4.93 U/L; 500 mg/kg WAG, 51.66±6.11 U/L). These results indicate that WAG administration at 500 mg/kg does not influence liver and kidney toxicity markers. The evaluation of hematological parameters is commonly used to diagnose side effects such as cancer, anemia, and wasting disease [36]. Thus, we investigated hematological parameters including RBC, HGB, Hct, MCV, MCH, and MCHC in WAG-administered mice. RBC levels in whole blood were not influenced by WAG administration (Figure 6(c)). Treatment with WAG statistically reduced HGB levels, but this alteration is imperceptible on body function (Figure 6(d)). Moreover, Hct, MCV, MCH, and MCHC levels were not changed by WAG administration (Figures 6(e), 6(f), 6(g), and 6(h)). These results indicate that WAG administration does not influence hematological parameters.

## 4. Discussion

In this study, we evaluated toxicity makers, hematological parameters, and effects of WAG administration in a CIA mouse model. The results showed that WAG improved the symptoms of RA by ameliorating the elevated serum levels of IL-6, TNF-*α*, and type II collagen IgG, the infiltration of inflammatory cells into the synovial membrane, and neutrophilia. Medicinal herbs used in traditional Korean and Chinese medicine have become controversial due to issues of safety [37, 38]. According to several reports, overdoses of various medicinal plant extracts have induced toxicity [39, 40]. A decrease in RBC in the blood is often associated with the development of malnutrition and anemia. Low HGB levels can be due to blood loss, and low Hct levels are often due to anemia [41, 42]. As previously mentioned, BUN and Cre serum levels are commonly used as indicators of kidney function. BUN and Cre levels are increased in cases of kidney dysfunction, including cases of diabetic nephropathy and nephrotoxicity [43]. Similarly, AST and ATL levels are increased in liver diseases such as liver cirrhosis and fatty liver disease [44]. In our results, BUN, Cre, ALT, and AST levels remained unchanged after WAG administration in DBA/1 mice. Moreover, treatment with WAG did not influence the hematological parameters RBC, HGB, and Hct. Thus, we suggest that liver and kidney toxicity markers and hematological parameters are not influenced by WAG administration in DBA/1 mice.

Previous studies have reported that blood inflammation indicators such as TNF-*α* and IL-6 levels are significantly increased in the synovial tissue and serum of RA patients and that these factors are involved in the destruction of the joints by inducing neutrophil migration and osteoclast maturation [45, 46]. Moreover, type II collagen IgG levels are greater in RA patients [47]. In this study, IL-6, TNF-*α*, and type II collagen IgG serum levels increased after the administration of bovine type II collagen and then diminished with 500 mg/kg WAG administration (Figures 5(a), 5(b), and 5(c)). In previous studies,* Acori Graminei* Rhizoma inhibited microglial neuroinflammation in an MPTP-induced mice model of Parkinson's disease [1], and a mixture containing* Acori Graminei* Rhizoma extract diminished the acetic acid-induced inflammatory response in rats [48]. These results support the effect of WAG administration on CIA animal model.

Immunological and pathological features in CIA animal models are similar to those in RA patients [49]. Our results indicate that administration of 500 mg/kg WAG decreased swelling and erythema of the hind limb and infiltration of inflammatory cells into the synovial membrane (Figure 4(c)). Neutrophils contribute to matrix destruction by regulating the secretion of MMP-8, MMP-9 and cathepsin G and involved RA pathology through the release of immunoregulatory and cytotoxic molecules, including cytokines and chemokines [50, 51]. As shown in Figures 2(a) and 2(b), neutrophil levels in the blood increased after CIA administration and decreased significantly after treatment with 0.2 mg/kg MTX or 500 mg/kg WAG in a CIA mouse models. These findings indicate that WAG acts as an antirheumatic agent in a CIA animal model. In order to fully understand this effect, further experiments are required to evaluate the signal transduction pathway* in vitro* and* in vivo*.

In conclusion, we found that WAG administration decreased swelling of the hind limb and infiltration of inflammatory cell into the synovial membrane. Moreover, we showed that serum levels of IL-6, TNF-*α*, and type II collagen IgG decreased after WAG administration, and we evaluated liver and kidney toxicity markers and hematological parameters in WAG-administered mice. Our findings provide convincing evidence that WAG administration diminishes symptoms of CIA in an animal models and that WAG has potential as a therapeutic agent for RA.

## 5. Conclusions

The results of this study indicate that WAG administration reduces elevated blood inflammation indicator including IL-6 and TNF-*α* with type II collagen IgG in serum. Moreover, the infiltration of inflammatory cells into the synovial membrane and swelling of the hind limb improved with WAG treatment. In addition, hematological parameters and toxicity marker of kidneys and liver were not changed by WAG administration. Thus, we suggested that WAG may act as therapeutic agent against rheumatoid arthritis. However, further experiments are required to explore how WAG influences the anti-inflammatory mechanism or inflammation signaling pathways including NF-kappaB or NACHT, LRR, and PYD domains-containing protein 3 (NLRP3).

## Figures and Tables

**Table 1:** LC-IT-TOF MS conditions of *Acori Graminei *Rhizoma extract.

HPLC condition			
Column	ACQUITY UPLC BEH C_18_ (2.1 × 150 mm, 1.7 *μ*m)		
Flow rate	0.21 mL/min		
Injection volume	1 *μ*L		
Column temperature	40°C		
	A: 0.1% formic acid in water		
	B: 0.1% acetonitrile		
Mobile phase	Time	A (%)	B (%)
	0	75	25
	20	50	50

MS condition			

Ionization mode	ESI, positive		
Capillary voltage (kV)	4.5		
CDL voltage (V)	10		
Detector voltage (kV)	1.67		
CDL temperature	200°C		
Heat block temperature	200°C		
Nebulizing gas	N_2_, 1.5 L/min		
Collision gas	Ar		

**Table 2:** Linear range, regression equation, and correlation coefficient of *β*-Asarone.

Compound	Linear range (ug/mL)	Regression equation	Correlation coefficient (R^2^)	LOD (ug/mL)	LOQ (ug/mL)
*β*-Asarone	12.0176-300.44	y= 31976.67x+10219.93	0.999	1.44	4.80

**Table 3:** Concentration of compound from extraction solvent.

Compound	Extraction solvent		Contents (mg/g)		Mean	SD	% RSD
		1	2	3			
*β*-Asarone	H_2_O	13.03	10.80	13.11	12.32	1.30	10.64
	30% MeOH	15.61	15.21	15.13	15.32	0.26	1.68
	50% MeOH	15.26	14.63	15.26	15.05	0.36	2.40
	70% MeOH	14.96	15.17	15.06	15.06	0.10	0.69
	MeOH	14.92	14.89	14.75	14.85	0.08	0.59

## Data Availability

The data used to support the findings of this study are available from the corresponding author upon request.
